# Nanoencapsulation of Gla-Rich Protein (GRP) as a Novel Approach to Target Inflammation

**DOI:** 10.3390/ijms23094813

**Published:** 2022-04-27

**Authors:** Carla S. B. Viegas, Nuna Araújo, Joana Carreira, Jorge F. Pontes, Anjos L. Macedo, Maurícia Vinhas, Ana S. Moreira, Tiago Q. Faria, Ana Grenha, António A. de Matos, Leon Schurgers, Cees Vermeer, Dina C. Simes

**Affiliations:** 1 Centre of Marine Sciences (CCMAR), Universidade do Algarve, 8005-139 Faro, Portugal; caviegas@ualg.pt (C.S.B.V.); nuna_araujo@hotmail.com (N.A.); jscarreira@ualg.pt (J.C.); pontes.jorge21@gmail.com (J.F.P.); amgrenha@ualg.pt (A.G.); 2 GenoGla Diagnostics, Centre of Marine Sciences (CCMAR), Universidade do Algarve, 8005-139 Faro, Portugal; 3 UCIBIO—Applied Molecular Biosciences Unit, Departamento de Química, and Associate Laboratory i4HB—Institute for Health and Bioeconomy, NOVA School of Science and Technology, Universidade NOVA de Lisboa, 2829-516 Caparica, Portugal; anjos.macedo@fct.unl.pt; 4 Algarve Biomedical Center Research Institute (ABC-RI), Universidade do Algarve, 8005-139 Faro, Portugal; mmvinhas@ualg.pt; 5 iBET—Instituto de Biologia Experimental e Tecnológica, 2780-157 Oeiras, Portugal; amoreira@ibet.pt (A.S.M.); tfaria@ibet.pt (T.Q.F.); 6 ITQB—Instituto de Tecnologia Química e Biológica António Xavier, Universidade Nova de Lisboa, 2780-157 Oeiras, Portugal; 7 Centro de Investigação Interdisciplinar Egas Moniz, Egas Moniz-Cooperativa de Ensino Superior CRL, 2829-511 Caparica, Portugal; apamatos@gmail.com; 8 Department of Biochemistry, Cardiovascular Research Institute, Maastricht University, 6229 HX Maastricht, The Netherlands; l.schurgers@maastrichtuniversity.nl; 9 Cardiovscular Research Institute CARIM, Maastricht University, 6229 HX Maastricht, The Netherlands; cees.vermeer@outlook.com

**Keywords:** nanoparticles, Gla-rich protein (GRP), chronic inflammatory diseases (CIDs), inflammation, vitamin K-dependent protein (VKDP)

## Abstract

Chronic inflammation is a major driver of chronic inflammatory diseases (CIDs), with a tremendous impact worldwide. Besides its function as a pathological calcification inhibitor, vitamin K-dependent protein Gla-rich protein (GRP) was shown to act as an anti-inflammatory agent independently of its gamma-carboxylation status. Although GRP’s therapeutic potential has been highlighted, its low solubility at physiological pH still constitutes a major challenge for its biomedical application. In this work, we produced fluorescein-labeled chitosan-tripolyphosphate nanoparticles containing non-carboxylated GRP (ucGRP) (FCNG) via ionotropic gelation, increasing its bioavailability, stability, and anti-inflammatory potential. The results indicate the nanosized nature of FCNG with PDI and a zeta potential suitable for biomedical applications. FCNG’s anti-inflammatory activity was studied in macrophage-differentiated THP1 cells, and in primary vascular smooth muscle cells and chondrocytes, inflamed with LPS, TNFα and IL-1β, respectively. In all these in vitro human cell systems, FCNG treatments resulted in increased intra and extracellular GRP levels, and decreased pro-inflammatory responses of target cells, by decreasing pro-inflammatory cytokines and inflammation mediators. These results suggest the retained anti-inflammatory bioactivity of ucGRP in FCNG, strengthening the potential use of ucGRP as an anti-inflammatory agent with a wide spectrum of application, and opening up perspectives for its therapeutic application in CIDs.

## 1. Introduction

Chronic inflammatory diseases (CIDs) are the most significant cause of death, with worldwide growing incidence, and are ranked as the greatest threat to human health by the WHO [1,2]. Amongst the wide spectrum of CIDs, cardiovascular diseases (CVD), chronic kidney disease (CKD), arthritis, osteoarthritis (OA), diabetes, neurodegenerative disorders and cancer are the leading causes of death and disability globally [1,2]. Inflammation, either under the conditions of a persistent low-grade inflammatory state implicated on the initiation, progression and outcomes in most CIDs, or as severe and acute pro-inflammatory reactions leading to multiorgan dysfunction, is a crucial event in this wide spectrum of complex diseases [1,2,3]. The lack of effective treatment leads to the wide consumption of anti-inflammatory drugs as the only option to lower inflammation and alleviate symptoms [2,3,4]. However, despite the wide availability of anti-inflammatory drugs, there is an urgent need for novel, safe and more efficient therapeutics to prevent and treat inflammation [4]. Moreover, in calcification-related CIDs such as atherosclerosis, CVD and OA, inflammation and pathological calcification are considered disease drivers, functioning in a positive feedback loop, influencing disease progression and outcomes [5,6]. In fact, there are common pathological processes involved in many of these diseases, such as dysregulated inflammatory pathways, pathological calcification and aberrant extracellular matrix (ECM) remodeling. The discovery of new drugs will enable us to improve one or all of these pathological processes, and could significantly contribute to developing or improving treatment options for some of these global health burdens.

Gla-rich protein (GRP), also known as upper zone of growth plate and cartilage matrix associated protein (UCMA) [7,8], is a circulating vitamin K-dependent protein (VKDP) shown to execute important functions in multiple processes associated with the development of CIDs, such as CVD, osteoarthritis, rheumatoid arthritis, and CKD [9,10,11,12,13,14], and has recently been proposed as a biomarker for vascular and valvular calcification (VC) and kidney dysfunction [15,16]. GRP is a potent inhibitor of pathological calcification, at the tissue and systemic levels, and is a protector of ECM degradation, with anti-inflammatory properties in monocytes, macrophages, chondrocytes and synoviocytes [5,9,10,11,14]. While GRP γ-carboxylation is crucial for its role as a calcification inhibitor, its anti-inflammatory activity has been shown to be independent of its γ-carboxylation status [5,9,10,11,14]. Importantly, increased GRP levels have been associated with tissue-beneficial effects, while GRP deficiency has been linked to disease states [7,8,9,10,11,12,13,14,15,16]. Its ability to act locally and systemically, with an exogenous and endogenous functional role, gives GRP promising and novel therapeutic properties. However, GRP’s limited solubility at physiological pH is a major challenge for its application in the biomedical field.

In this context, nanotechnology, and in particular nanoparticles (NP), have been proposed as valuable vehicles for efficient drug transport by protecting the bioactive elements from degradation, thus increasing their bioavailability, efficacy, specificity, uptake and targeting ability [17]. Among the many matrixes and methodologies currently available to produce nanoparticles (NPs), chitosan (CS)-based NPs have become one of the most popular nanocarriers for a variety of drugs, such as proteins, peptides, nucleic acids and other bioactives [18,19,20,21,22,23,24]. This is mainly due to CS’s favorable biological properties, such as biodegradability, biocompatibility and low toxicity, as well as its bactericidal, fungicidal, anticancer and immunomodulatory properties [18,19,20]. Additionally, CS has mucoadhesive properties and promotes macromolecules’ permeation through epithelia, which is key for drug delivery purposes [21,25,26]. CS/tripolyphosphate (CS/TPP) NPs produced by ionotropic gelation are amongst the most widely studied CS/NPs, and have been suggested to be suitable for many biological applications, due to their mild and aqueous processing conditions, which include non-toxic reagents and low energy requirements [20,23,24,26,27,28]. 

In this work, we developed a new nanoparticle formulation containing non-carboxylated GRP (ucGRP), which comprises fluorescein-labeled CS/TPP nanoparticles produced by ionotropic gelation, and tested its anti-inflammatory properties in macrophage-differentiated THP1 (THP1-MoM), in human primary chondrocytes and VSMCs, as relevant cell systems involved in such CIDs as osteoarthritis and CVD.

## 2. Results

### 2.1. Characterization of Fluorescein-Labeled Chitosan (FC)- Tripolyphosphate GRP Nanoparticles (FCNG)

Fluorescein-labeled chitosan (FC) was obtained by chemical addition of the fluorescein carboxylic acid groups to the primary amine groups of the D-glucosamine residues of chitosan, mediated by 1-ethyl-3-(3-dimethylaminopropyl) carbodiimide hydrochloride (EDAC), yielding 84.5% FC. Nanoparticles with and without GRP (FCNG and FCNP, respectively) were prepared by ionic gelation, following a previously described methodology [29,30]. The characterization of FCNP and FCNG was performed by nanoparticle tracking analysis (NTA) and dynamic light scattering (DLS) (Table 1).

**Figure 1:**
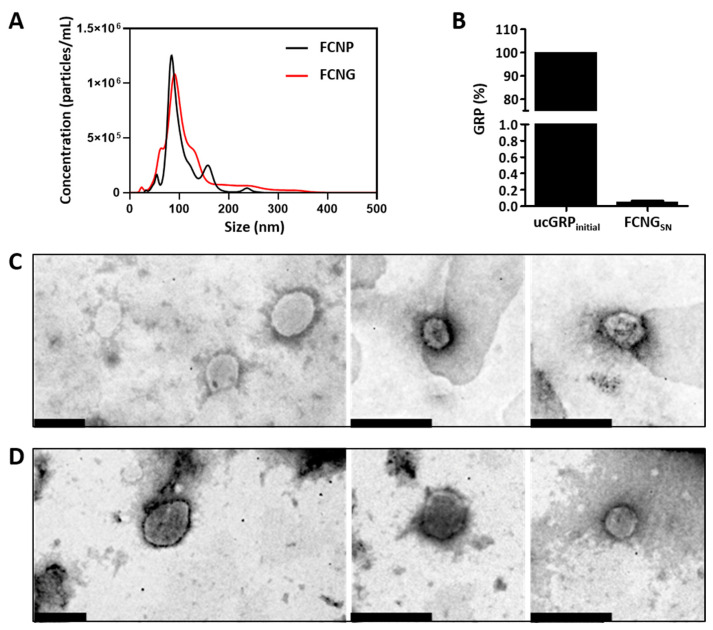
FCNP and FCNG size distribution, ucGRP incorporation and morphology. (**A**) Representative analysis of FCNP and FCNG by nanotracking analysis (NTA), showing the concentration of particles as a function of size. (**B**) Percentage of ucGRP initially used for FCNG synthesis (ucGRP_initial_), and in the supernatants after separation of pelleted nanoparticles (FCNG_SN_), representing non-incorporated ucGRP, determined by ELISA. Data are representative of six independent experiments. (**C**,**D**) Ultrastructural characterization of FCNP (**C**) and FCNG (**D**) by transmission electron microscopy (TEM). Scale bar of 200 nm.

Considering the relative amounts of particles by size distribution, NTA analysis demonstrated the presence of 100% of both FCNP and FCNG between 0 and 400 nm, and contents of 92.8 ± 9.3% and 90 ± 4.7% 200 nm for FCNP and FCNG, respectively (Figure 1A). These distributions were found to be mainly unimodal for FCNP and FCNG, highlighting a major population with a peak of 88 ± 24 nm for FCNP and of 107 ± 34 nm for FCNG, although the former presented a slightly broader distribution (Figure 1A). Total particle concentration determined by NTA was found to be constant between FCNP ((8.8 ± 3.6) × 10^9^ particles/mL) and FCNG ((14.5 ± 3.4) × 10^9^ particles/mL), without statistically significant differences. DLS analysis revealed a mean particle size of 130 ± 45 nm for FCNP and 155 ± 38 nm for FCNG, which is consistent with the NTA determinations, with a polydispersity index (PDI) of 0.33 ± 0.07 and 0.39 ± 0.06, respectively (Table 1). The zeta potential was positive for both FCNP and FCNG, and slightly decreased from FCNP, at 37 ± 2 mV, to FCNG, at 28 ± 7 mV (Table 1). All parameters were non-statistically different between FCNP and FCNG. 

The association efficiency (AE) of the GRP in FCNGs was calculated based on the quantification by ELISA of the GRP present in the supernatant after FCNGs synthesis, representing the fraction of GRP not encapsulated (Figure 1B), relative to the quantity of protein initially used. The results show a high degree of GRP encapsulation with an association efficiency (AE) of 99.8 ± 0.07 (Table 1), indicating effective GRP incorporation. Ultrastructural analyses of FCNP (Figure 1C) and FCNG (Figure 1D), by transmission electron microscopy (TEM), have revealed small nanoparticles with spherical morphologies, primarily varying from 100 to 250 nm in both FCNP and FCNG.

### 2.2. GRP Release Profile from FCNG

**Figure 2:**
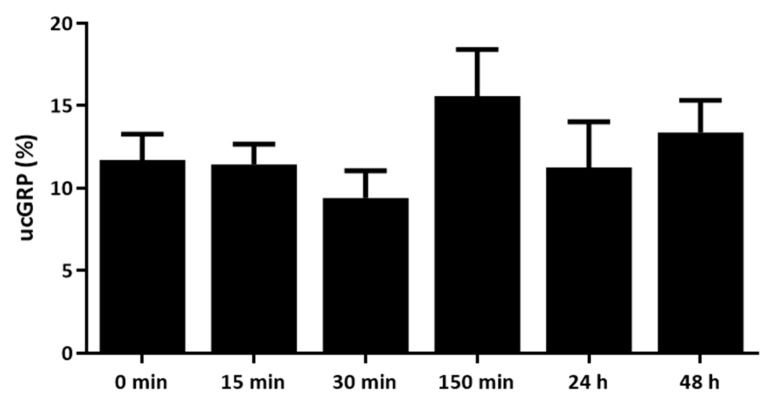
Levels of ucGRP released from FCNG during a 48 h study. ucGRP in cell culture media (RPMI) was measured by ELISA, at different time points from 0 min to 48 h. The data are representative of three independent experiments, and the differences are statistically non-significant.

In order to explore the stability of FCNG for further use in cell culture functional assays and future potential therapeutic applications, the rate of GRP release from FCNG in cell culture media was assessed over time. For this, FCNG was resuspended and maintained in conditions mimicking the physiological environment of the THP-1 cell culture for different time periods. Under these conditions, the release rate of GRP from FCNG, determined through GRP quantification by ELISA, was fairly constant through time, with 10–15% release after 48 h of FCNG suspension (Figure 2). 

### 2.3. Anti-Inflammatory Effect of FCNG in THP-1 MoM Cells Is Mediated by Increased Intracellular and Extracellular GRP Levels and Downregulation of Pro-Inflammatory Mediators

**Figure 3:**
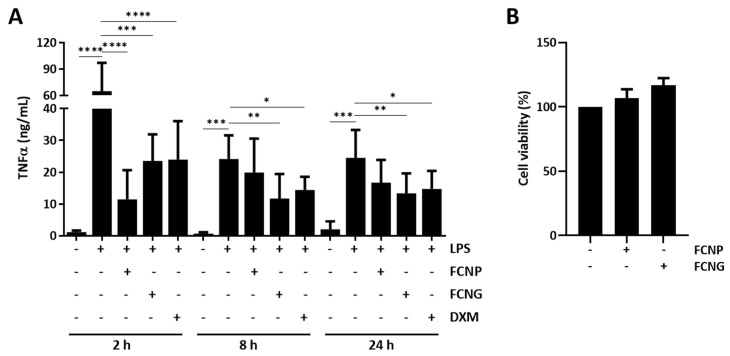
Anti-inflammatory effect and toxicity of FCNP and FCNG in LPS-stimulated THP-1 macrophages (THP-1-MoM). (**A**) The evaluation of the inflammatory marker TNFα was performed by ELISA in the cell culture media of THP-1 MoM treated for 2 h, 8 h or 24 h with (11.7 ± 4.5) × 10^9^ particles/mL of FCNP or FCNG, and then stimulated with LPS (100 ng/mL) for a further 24 h. Dexametasone (DXM) (2 μM) was used as a positive anti-inflammatory control and non-stimulated cells were used as controls for LPS stimulation. (**B**) Viability of THP-1-MoM exposed to (11.7 ± 4.5) × 10^9^ particles/mL of FCNP or FCNG for 48 h. Data are representative of three independent experiments, and presented as mean ± SD. Two-way ANOVA and multiple comparisons were achieved with the Dunnett’s test and are presented relative to the LPS-stimulated cells. Statistical significance was defined as *p* ≤ 0.05 (*), *p* ≤ 0.01 (**), *p* ≤ 0.001 (***) and *p* ≤ 0.0001 (****).

The functionality of FCNG on the modulation of in vitro inflammatory responses was evaluated in LPS-stimulated THP-1 MoM cells pre-treated with FCNP and FCNG for different times, followed by 24 h LPS stimulation. LPS was selected as the THP-1 MoM inflammatory stimulus, since it has been shown to induce not only a strong pro-inflammatory reaction, but also a low-grade inflammatory condition associated with several CIDs, opening the range of potential FCNG applications [29]. The anti-inflammatory effect was analyzed by measuring the levels of TNFα released into the cell culture media. The results show decreased TNFα levels in FCNG-treated cells at all durations tested, with the highest effect at 8 h that lasted until 24 h of pre-treatment (Figure 3A). An anti-inflammatory effect of the FCNP was also observed, although this was restricted to the 2 h of nanoparticle pre-treatment, at the inflammation peak response (Figure 3A). Cell proliferation assays have demonstrated that FCNP and FCNG application at the concentrations used in this study did not affect THP-1 MoM cell viability (Figure 3B).

**Figure 4:**
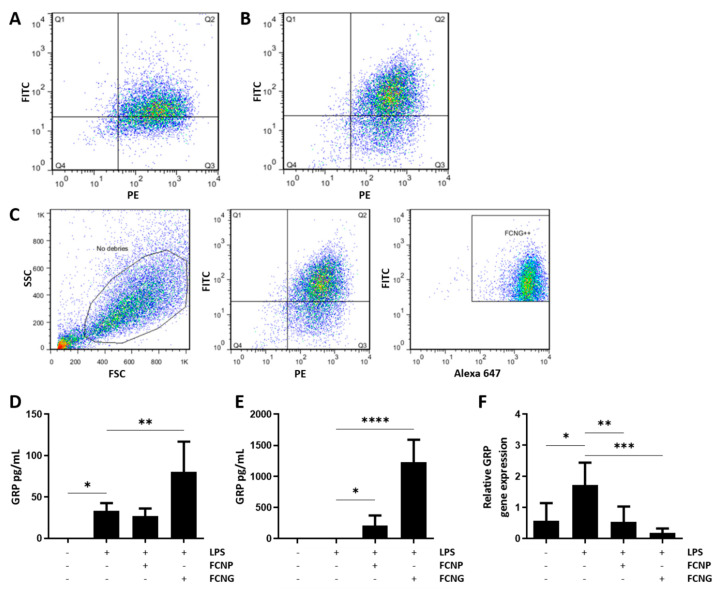
Binding/uptake of FCNP and FCNG by THP1-MoM cells. (**A**–**C**) Flow cytometry analysis of FCNP and FCNG in THP1-MoM cells. (**A**,**B**) Dot plots of THP-1 MoM cells exposed to (11.7 ± 4.5) × 10^9^ particles/mL of FCNP (**A**) and FCNG (**B**) for 2 h. The Q2 quadrant represents cells that have, simultaneously, fluorescein-labeled nanoparticles and the THP-1 MoM marker labeled with PE (double positive for FITC and PE). Q2 is 67.1% for FCNP (**A**) and 73.7% for FCNG (**B**). (**C**) Gating strategy used for the analysis of FCNG in THP1-MoM cells. The first plot shows the debris exclusion in the side scatter (SSC) vs. the forward scatter (FSC). In the second plot, the double positive population for fluorescein-labeled nanoparticles and the THP-1 MoM marker was gated (73.7% are double positive for FITC and PE). Finally, the last plot shows the selection of the population of interest, FCNG++, which represents THP-1 MoM cells with fluorescein-labeled nanoparticles containing GRP labeled with Alexa 647 (73.3% are triple positive for FITC, PE and Alexa 647). (**D**,**E**) Quantification of GRP present in THP1-MoM cell protein extracts (**D**) and in the cell culture media (**E**) by ELISA, after pre-treatments with (11.7 ± 4.5) × 10^9^ particles/mL of FCNP and FCNG for 24 h, followed by stimulation with LPS (100 ng/mL) for an additional 24 h. Non-stimulated cells were used as controls for LPS stimulation. (**F**) Relative GRP gene expression determined by quantitative polymerase chain reaction (qPCR) of experiments described in (**D**,**E**). Data in (**D**–**F**) are representative of three independent experiments and presented as mean ± SD. Two-way ANOVA and multiple comparisons were achieved with the Dunnett’s test, and presented relative to the LPS-stimulated cells. Statistical significance was defined as *p* ≤ 0.05 (*), *p* ≤ 0.01 (**), *p* ≤ 0.001 (***) and *p* ≤ 0.0001 (****).

To evaluate the THP-1 MoM binding/uptake of FCNP and FCNG, flow cytometry studies were performed after 2 h of treatments with nanoparticles. A triple-stained system was designed and optimized to simultaneously detect the fluorescein-based nanoparticles through FITC, GRP was detected through ALEXA647, and PE was used for the extracellular CD11b membrane labeling. The results show two conjoint positive signals for the presence of fluorescein (FITC) and cell membrane labeling (PE), demonstrating that 67.1% of the cells exposed to FCNP (Figure 4A), and 73.7% of the cells exposed to FCNG (Figure 4B), were positive for fluorescein-labeled NPs. The presence of FCNG was further confirmed by the high-intensity fluorescence of the three different fluorophores, corresponding to fluorescein (FITC), cell membrane labeling (PE) and GRP (ALEXA 647), demonstrating that 73.3% of the FCNG are present in the THP1- MoM cells (Figure 4C), and confirming the presence of ucGRP in this formulation.

In addition, intracellular and extracellular GRP levels were determined in THP1-MoM cell extracts and conditioned media from cells pre-treated with FCNP and FCNG for 24 h, followed by 24 h LPS stimulation. The results show increased intracellular GRP levels with LPS treatment (Figure 4D), consistent with previous results showing a GRP upregulation during inflammation [5]. Treatments with FCNP have no effect on intracellular GRP protein levels, while treatments with FCNG result in a strong increase in intracellular GRP (Figure 4D). GRP quantification in the cell culture media showed high levels of extracellular GRP in THP1-MoM cells treated with FCNG (Figure 4E). Interestingly, gene expression analysis revealed that FCNG treatments induce a strong downregulation of GRP gene expression (Figure 4F). 

These results strongly indicate that the overall increase in levels of GRP in THP1-MoM cells treated with FCNG results from the exogenously added GRP nanoparticles, and that the anti-inflammatory effect of FCNG is mediated not only by increased extracellular GRP levels, but also by the increased intracellular GRP levels most probably resulting from FCNG uptake by THP1-MoM cells. 

**Figure 5:**
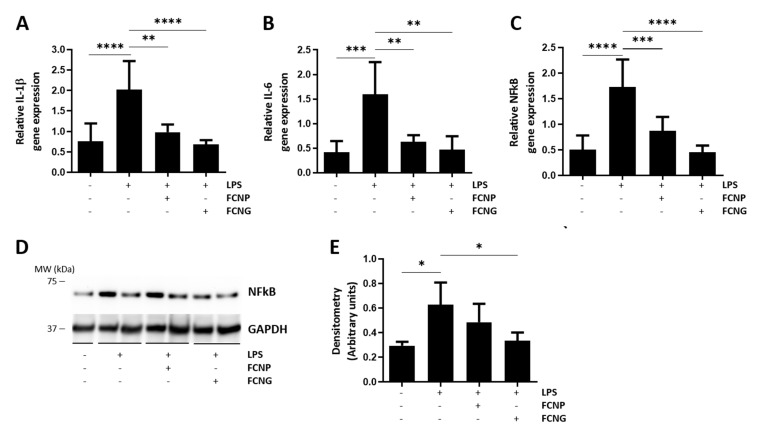
The anti-inflammatory activity of FCNG in THP1-MoM cells is mediated by downregulation of pro-inflammatory cytokines. (**A**–**C**) Gene expression analysis of IL-1β (**A**), IL-6 (**B**) and NFkB (**C**) by qPCR of THP1-MoM cells pre-treated with (11.7 ± 4.5) × 10^9^ particles/mL of FCNP and FCNG for 24 h, followed by stimulation with LPS (100 ng/mL) for an additional 24 h. Non-stimulated cells were used as controls for LPS stimulation. (**D**,**E**) Total protein extracts of THP1-MoM cells treated as described in (**A**–**C**) were analyzed by Western blot to detect NFkB. The positions of relevant molecular mass markers (kDa) are indicated on the right side and GAPDH was used as the loading control. (**E**) Quantification of NFkB levels was performed by densitometry using ImageJ software, and is presented relatively to the GAPDH loading control as arbitrary units. Data are representative of three independent experiments and presented as mean ± SD. Two-way ANOVA and multiple comparisons were performed with the Dunnett’s test, and are presented relative to the LPS-stimulated cells. Statistical significance was defined as *p* ≤ 0.05 (*), *p* ≤ 0.01 (**), *p* ≤ 0.001 (***) and *p* ≤ 0.0001 (****).

In addition to the effect of decreasing TNFα levels (Figure 3A), the gene expression analysis of THP1-MoM cells treated with FCNP and FCNG for 24 h, followed by 24 h LPS stimulation, revealed that both FCNP and FCNG are able to downregulate IL-1β, IL-6, and NFkB gene expression, although the effect is stronger with FCNG treatment (Figure 5A–C). Western blot analysis has shown a significant decrease in NFkB protein levels in THP1-MoM cells treated with FCNG (Figure 5D,E). These results reinforce the anti-inflammatory activity of FCNG, which is mediated by decreased levels of pro-inflammatory cytokines and the NFkB that is well-known as a key player in signaling pro-inflammatory reactions.

### 2.4. Anti-Inflammatory Potential of FCNG in Human Primary Vascular Smooth Muscle Cells (VSMCs)

**Figure 6:**
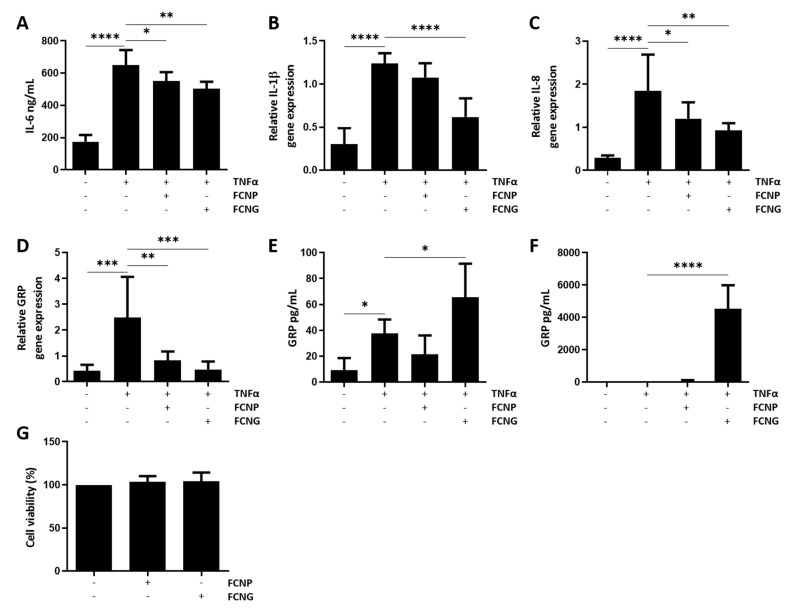
Anti-inflammatory activity of FCNG in primary human VSMCs. VSMCs were pre-treated with (11.7 ± 4.5) E + 9 particles/mL of FCNP and FCNG for 24 h, followed by stimulation with TNFα (20 ng/mL) for an additional 24 h, then analyzed for levels of IL-6 present in the cell culture media by ELISA (**A**) and levels of gene expression of IL-1β (**B**), IL-8 (**C**) and GRP (**D**) by qPCR. (**E**,**F**) Quantification of GRP present in VSMCs protein extracts (**E**) and in the cell culture media (**F**) by ELISA. Non-stimulated cells were used as controls for TNFα stimulation. (**G**) Viability of VSMCs exposed to (11.7 ± 4.5) × 10^9^ particles/mL of FCNP or FCNG for 48 h. Data are representative of three independent experiments and presented as mean ± SD. Two-way ANOVA and multiple comparisons were performed with the Dunnett’s test, and are presented relative to the TNFα-stimulated cells. Statistical significance was defined as *p* ≤ 0.05 (*), *p* ≤ 0.01 (**), *p* ≤ 0.001 (***) and *p* ≤ 0.0001 (****).

Since GRP has been shown to be involved in the downregulation of pro-inflammatory mediators in VSMCs during the calcification induced by calciprotein particles (CPPs) isolated from CKD stage 5 patients [11], the effect of FCNG in the inflammatory response of VSMCs stimulated with TNFα was evaluated. This selection of TNFα as the inflammatory stimulus to VSMCs was based on its suggested pivotal role in vascular dysfunction, contributing to the pathogenesis of many CVDs, and its capacity to promote vascular calcification in vitro [30,31]. VSMCs pre-treated for 24 h with both FCNP and FCNG followed by 24 h of TNFα stimulation showed decreased levels of IL-6 relative to cells treated only with TNFα, but this was clearly more significant with FCNG (Figure 6A). Similarly, gene expression analysis demonstrated a more significant downregulation of IL-1β and IL-8 in VSMCs pre-treated with FCNG relative to FCNP (Figure 6B,C). VSMCs inflammation induced by TNFα results in increased GRP gene expression, while pre-treatments with both FCNP and FCNG induced GRP downregulation (Figure 6D). Increased intracellular GRP accumulation is also observed with TNFα treatments, although higher levels of intracellular and extracellular GRP protein are achieved with FCNG treatments (Figure 6E,F). Intra- and extracellular GRP protein levels were not affected by FCNP treatments (Figure 6E,F). GRP upregulation and increased endogenous protein levels, concomitant with increased levels of pro-inflammatory cytokines, suggest the involvement of endogenous GRP in the inflammatory response of VSMCs. GRP downregulation together, with increased intra- and extracellular protein levels following FCNG treatments, strongly indicate FCNG as the exogenous GRP source. Overall, these results further confirm the anti-inflammatory activity of FCNG, thus widening the potential therapeutical application of FCNG in cardiovascular disease-related inflammation. Cell proliferation assays have demonstrated that applying FCNP and FCNG at the concentrations used in this study did not affect the VSMCs’ viability (Figure 6G).

### 2.5. Anti-Inflammatory Potential of FCNG in Human Primary Articular Chondrocytes

**Figure 7:**
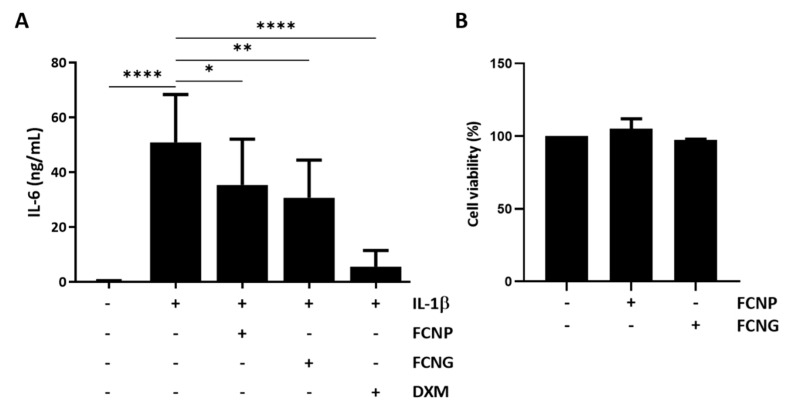
Anti-inflammatory activity of FCNG in primary human articular chondrocytes. (**A**) Chondrocytes were pre-treated with (11.7 ± 4.5) × 10^9^ particles/mL of FCNP and FCNG for 24 h, or dexametasone (DXM, 2 µM), followed by stimulation with IL-1β (10 ng/mL) for an additional 24 h, and analyzed for levels of IL-6 present in the cell culture media by ELISA. Non-stimulated cells were used as controls of IL-1β stimulation. (**B**) Viability of articular chondrocytes exposed to (11.7 ± 4.5) × 10^9^ particles/mL of FCNP or FCNG for 48 h. Data are representative of three independent experiments and are presented as mean ± SD. Two-way ANOVA and multiple comparisons were performed with the Dunnett’s test, and are presented relative to the IL-1β-stimulated cells. Statistical significance was defined as *p* ≤ 0.05 (*), *p* ≤ 0.01 (**), and *p* ≤ 0.0001 (****).

The anti-inflammatory activity of FCNG was also tested in human primary chondrocytes pre-treated with FCNG and FCNP for 24 h, followed by 24 h of IL-1β stimulation. IL-1β was selected as the chondrocyte inflammation stimulus since it has been shown to be a highly potent inducer of cartilage degradation, and suggested as an important mediator involved in the pathogenesis of OA [32]. The levels of IL6 released into the cell culture media were used to evaluate the inflammatory response. In chondrocytes, both FCNP and FCNG were able to decrease IL6 levels, although the effect was more significant with the FCNG treatments (Figure 7A). Cell proliferation assays demonstrated that FCNP and FCNG at the concentrations used in this study did not affect articular chondrocytes viability (Figure 7B). These results indicate an anti-inflammatory functional activity of FCNG in chondrocytes, opening up the way for its future therapeutic applications in articular-associated inflammatory diseases, such as OA.

## 3. Discussion

In this study, we developed a novel ucGRP nanoparticle formulation (FCNG), using the well-described drug delivery system of CS-TPP nanoparticles. We show that FCNG is not cytotoxic, and functions as an anti-inflammatory agent in human macrophage-derived THP1 cells, primary VSMCs and articular chondrocytes. FCNG’s anti-inflammatory action was observed with the different pro-inflammatory agents used to induce inflammation, particularly LPS in THP-1 MoM, TNFα in VSMCs, and IL-1β in articular chondrocytes, representing the relevant tissue-associated inflammatory stimuli involved in a wide spectrum of CIDs [29,30,31,32]. Although we cannot infer at this point FCNG’s mechanism of action in these different cell-specific inflammatory-stimulated conditions, the overall outcome of decreasing pro-inflammatory mediators open up new perspectives for the therapeutic application of FCNG in several CIDs.

Although the beneficial effects of ucGRP have been described at multiple levels, its potential use as a therapeutic agent is hindered by its low solubility at physiological pH. Our strategy to overcome this is based on the nanoencapsulation of ucGRP using CS-TPP nanoparticles, taking advantage of the widely reported biological properties of CS, combined with well-described procedures for CS-TPP nanoparticle production and protein encapsulation [19,20,21,23,24]. In fact, not only are chitosan-based nanoparticles among the most intensively studied nanosystems for drug delivery in biomedical applications, but CS-TPP nanoparticles have also been widely described as being capable of incorporating and releasing a variety of drugs, such as proteins, peptides, vitamins, and other bioactive compounds [20,22,23,24,28,33,34,35,36]. Importantly, CS and CS-derived NPs have been shown to have anti-inflammatory properties by decreasing pro-inflammatory cytokine production, although the immunomodulatory functions of chitosan are still unclear [37,38,39,40]. In concordance, our results show the anti-inflammatory activity of FCNP in all in vitro cell systems tested, strengthening the beneficial use of the CS excipient by fulfilling requirements and adding a therapeutic effect, as a complement to GRP’s anti-inflammatory activity. 

The characterization of the CS–TPP nanoparticles produced in the present study revealed their nanoscale nature, at around 120–150 nm, which is consistent with the majority of the reports of CS–TPP nanoparticles produced by similar methods, ranging from 40 to 250 nm [20,22,23,24,28,33,34,35,36]. This wide range can be explained by different factors known to influence nanoparticles size, such as chitosan concentrations and molecular weight, the degree of CS acetylation, and the CS/TPP molar ratio [20,27]. In our study, we used fluorescein-labeled CS to produce FCNP and FCNG formulations, which might differ in size from unlabeled CS/TPP nanoparticles, although similar fluorescein-labeling has been demonstrated not to interfere with CS nanoparticles’ properties [21,25]. The advantages of fluorescein-labeling nanoparticles include their easy detection due to intense fluorescence emission, allowing them to be quickly tracked intra- and extracellularly, which is extensively used as a tool to study nanoparticles association with cells in biomedical and pharmacological applications [21]. This feature makes FCNG a powerful tool for further applications at both the basic research and biomedical levels. The mean size of FCNG nanoparticles was shown to not significantly differ from that of FCNP, despite the high ucGRP association efficiency, which was expected due to the small molecular weight of the protein and the relatively low ucGRP concentrations used in this study. Additionally, FCNG’s broader particle distribution indicates a continuum of particles slightly differing in size, which might reflect different quantities of ucGRP molecules encapsulated, and indicates a non-saturation point of ucGRP in NPs. Nevertheless, the selected method of ucGRP incorporation during particle formation—by adding ucGRP to FC followed by TPP addition—resulted in a high degree of ucGRP association, in concordance with previous reports showing the higher loading efficiency of this incorporation method [20,23]. The successful incorporation of ucGRP into the FCNG formulation was further confirmed by flow cytometry. PDI values of around 0.3–0.4 for FCNP and FCNG did not significantly differ, and suggest suitability for a biomedical or pharmaceutical application. The positive zeta potential of both FCNP and FCNG, reflecting the cationic nature of CS, was found to be slightly, but not significantly, lower in FCNG as compared to FCNP—+37 mV compared to +28 mV—although still in the acceptable range for stable colloidal dispersions [41]. 

Overall, our new data show that ucGRP can be efficiently incorporated into CS–TPP nanoparticles, giving rise to a nanoparticle formulation suitable for several applications, including biomedical research and engineering.

The functionality of the FCNG formulations in the present study was due to their immunomodulatory activity, enabling us not only to evaluate the bioactivity of ucGRP in FCNG, but also to obtain new insights into the potentialities of the ucGRP anti-inflammatory agent. While GRP γ-carboxylation has been shown to be essential for calcification inhibition [9,10,11,14], the non carboxylated GRP (ucGRP) used in the FCNG formulation has been shown to function as an anti-inflammatory agent by decreasing pro-inflammatory reactions in target cells, such as THP1-MoM cells, chondrocytes and synoviocytes [5,9]. Our results demonstrate that FCNG is able to decrease the pro-inflammatory cytokines and inflammation mediators in THP1-MoM and chondrocyte in vitro cell systems. The downregulation of NFkB gene expression and decreased protein accumulation, together with the decreased levels of TNFα, IL-1β and IL-6, with FCNG treatment, clearly demonstrate the retention of GRP anti-inflammatory activity. Moreover, in the present work, we extended our studies to evaluate the effects of ucGRP anti-inflammatory activity in VSMCs using treatments with FCNG. We previously demonstrated that GRP is involved in the downregulation of pro-inflammatory mediators in VSMCs during the calcification induced by calciprotein particles (CPPs) isolated from CKD stage 5 patients [11]. While these results clearly indicate a role for GRP as a mediator between calcification and inflammation, its function in the classical activation of VSMCs inflammation with cytokines is still unknown. Our results demonstrate a typical pro-inflammatory response of VSMCs upon TNFα stimulation, increasing levels of IL-6, IL-1β and IL-8 pro-inflammatory cytokines along with the upregulation and increased production of GRP. This increase in GRP levels is similar to the response of the LPS-stimulated THP1-MoM previously reported [5] and confirmed in the present study, and strongly indicates GRP as an important agent mediating pro-inflammatory reactions in multiple cells. Treatments of VSMCs with FCNG clearly reduced the production of these pro-inflammatory cytokines, extending the suitability of using FCNG as an anti-inflammatory agent in cytokine-induced vascular inflammation. 

These results are particularly relevant in the context of several chronic inflammatory diseases, such as CVD and OA, which share an intricate pathological inflammation–calcification cycle contributing to disease initiation and progression [5,6]. The dual functionality of GRP as an anti-calcifying and anti-inflammatory agent, combined with a suitable system for GRP delivery such as FCNG, can significantly contribute to developing or improving treatment options for some of these global health burdens.

Although the mechanism of action of GRP in relation to inflammation remains unclear, GRP has been shown to function at both intra- and extracellular sites in the inhibition of ectopic calcification. The major advantages of GRP nano-encapsulation are expected to enable it to deliver GRP to both cellular environments. Our data showing the presence of FCNG in THP1-MoM cells after 2 h of incubation, and the increased intra- and extracellular GRP levels after 24 h in both THP1-MoM and VSMCs downregulating GRP expression, strongly indicate sustained and prolonged ucGRP delivery through FCNG formulation. This is consistent with results showing low GRP leaching from nanoparticles when incubated only in cell culture media, probably reflecting the protein desorption from the nanoparticles’ surface [20], indicating that the majority of GRP is entrapped into FCNG, thus conferring biological stability. Although our present data do not allow us to differentiate between intracellular and surface-located FCNG, CS and particularly CS NPs are known to be efficiently internalized by cells, mainly by nonspecific electrostatic forces that enhance the paracellular permeability of opening tight junctions, by adsorptive endocytosis partially mediated by a clathrin-mediated process, and phagocytosis in the particular case of macrophages [21,26]. It is possible that internalized FCNG further releases GRP through diffusion from the pores of the polymer network, and/or degradation and erosion of the nanoparticle. Following internalization, CS NPs to rapidly and partially dissociate within lysosomes, through enzymatic degradation [42,43]. This can partially explain the rapid and strong effect of FCNP in the anti-inflammatory response of THP1-MoM cells after 2 h, with a longstanding effect of FCNG seen up to 24 h, probably reflecting GRP’s sustained release and functionality. 

Overall, we have demonstrated that GRP’s incorporation into CS–TPP nanoparticles results in a novel, stable and biodegradable formulation of ucGRP with physiochemical properties suitable to several biomedical applications, and with the retained functionality of GRP’s anti-inflammatory activity in the different human in vitro cell systems tested. This brings more advantages, and strengthens the wide range of GRP applications in several inflammation-related diseases, opening the way to further GRP nanoparticle improvements that will ultimately enable an effective anti-inflammatory therapy. 

## 4. Materials and Methods

### 4.1. Production and Quantification of Recombinant Human Non-Carboxylated GRP (ucGRP)

Recombinant human non-carboxylated GRP (ucGRP) was produced in the *E. coli* system and purified by affinity chromatography (HisTrap HP Column, GE Healthcare) followed by Reverse Phase–High Performance Liquid Chromatography (RP-HPLC), as previously described [44]. GRP quantification was performed using a specific sandwich ELISA for the quantification of total GRP, as described [11]. 

### 4.2. Chitosan Labeling with Fluorescein (FC) 

The labeling of chitosan (CS) with fluorescein was performed according to a previously established procedure [25]. Briefly, 250 mg of deacetylated CS (low molecular weight, deacetylation degree 75–80%) (Sigma Aldrich, Burlington, MA, USA) was dissolved in 15 mL of 1% (*v*/*v*) acetic acid solution, at room temperature. Fluorescein sodium salt (Sigma Aldrich, Burlington, MA, USA) (10 mg) was dissolved in 1 mL of ethanol (96%), and 7.5 mg of 1-ethyl-3-(3-dimethylaminopropyl) carbodiimide (EDAC) (Sigma Aldrich) was dissolved in 4 mL of Milli-Q water. The fluorescein solution was carefully added to the chitosan solution under stirring, followed by the addition of the EDAC solution. The mixture was stirred overnight at room temperature, protected from light, followed by dialysis against water using a 2000 molecular weight cut-off tubing (Sigma-Aldrich) over 3 days, with constant medium changes. The entire dialyzed solution was freeze-dried, and the process yield (PY) was calculated as follows Equation (1):

PY (%) = CS weight/Total solids (CS + Fluorescein) weight × 100(1)

### 4.3. Preparation of Fluorescein-Labeled Chitosan (FC)/TPP (FCNP) and FC/GRP/TPP (FCNG) Nanoparticles

Fluorescein-labeled chitosan/TPP nanoparticles were prepared by ionotropic gelation through the electrostatic interaction of the positively charged amino groups of CS with negatively charged phosphate groups of TPP anion, using a previously described methodology [33,34]. Briefly, CS was dissolved in 1% acetic acid (*v*/*v*) and TPP (Sigma Aldrich) was dissolved in Milli-Q water at room temperature, to obtain solutions of 1 mg/mL (*w*/*v*) and 0.714 mg/mL (*w*/*v*), respectively. FCNP were formed by the addition of 0.8 mL of the TPP solution to 2 mL of the CS solution to reach the theoretical FC/TPP ratio of 3.5/1 (*w*/*w*), under constant magnetic stirring at room temperature. FCNG was similarly prepared, and 1 µg of ucGRP was added to 2 mL of CS (1 mg/mL), and then allowed to react with 0.8 mL TPP (0.714 mg/mL). Nanoparticles were concentrated by centrifugation at 16,000× *g* at 15 °C, for 30 min. The supernatants were used for the quantification of GRP non-incorporated into FCNG, and nanoparticles were resuspended either in Milli-Q water or cell culture media for physicochemical characterization and in vitro cell functional assays, as described below.

### 4.4. Characterization of FCNP and FCNG, Physicochemical Properties and Morphology

The concentrations and size distributions of both FCNP and FCNG particles were determined using the Nanoparticle Tracking Analysis NTA technique on the NanoSight NS300™ (Malvern Instruments, Worcestershire, UK) equipment. The samples were initially solubilized in MilliQ water to a final volume of 2 mL, and then diluted 100-fold with MilliQ water. Each sample was analyzed 3 times (*n* = 3) with independent dilutions. Capture settings (shutter and gain) were adjusted manually for each analysis and all steps were carried out at room temperature. Sample videos were analyzed with the NTA 2.3 Analytical software.

FCNP and FCNG were characterized regarding their size, polydispersity index (PDI) and zeta potential on freshly prepared samples by photon correlation spectroscopy and laser Doppler anemometry, respectively, using a Zetasizer^®^ NanoZS (Malvern Instruments, Malvern, UK). The characterization was performed in an electrophoretic cell, in which a 20 µL aliquot of nanoparticles was diluted in 1 mL of either cell culture media (RPMI 1640) for size and PDI determinations, or ultrapure water for zeta potential measurements. Size and PDI were determined with a detection angle of 173°, at 25 °C, and zeta potential was calculated from the mean electrophoretic mobility values. Three batches each of FCNP and FCNG were analyzed in triplicate (*n* = 3). 

Morphological analysis was performed by the transmission electron microscopy (TEM) of negative-stained FCNP and FCNG. Freshly prepared NPs were resuspended in ultrapure water and adsorbed onto formvar grids. Samples were stained with 1.5% aqueous uranyl acetate for TEM image acquisition in a JEOL 1200EX transmission electron microscope. 

### 4.5. Determination of ucGRP Association Efficiency (AE) 

ucGRP association efficiency (AE) refers to the amount of protein associated with FCNG, expressed as a percentage of the total amount of ucGRP added in the process. The amount of free non-associated ucGRP was determined by ELISA in the supernatants of the reaction medium after separation by centrifugation (16,000× *g*, 30 min, 15 °C). ucGRP AE was determined from three independent FCNG preparations (*n* = 6). 

The association efficiency (AE) of ucGRP was calculated as follows Equation (2):

AE (%) = (Total ucGRP amount − Free ucGRP amount)/Total ucGRP amount × 100(2)

### 4.6. In Vitro Release of ucGRP from FCNG

In vitro studies of the release of ucGRP from FCNG nanoparticles were performed by resuspending the FCNG pellets in 2 mL of RPMI, at 37 °C and 5% of CO_2_ atmosphere, for appropriate time intervals of between 0 min and 48 h. At the end of each time point, equal volumes were evaluated for ucGRP content. For this, the samples were centrifuged for 30 min, at 15 °C and 16,000× *g*, and the ucGRP released was quantified in the supernatant by ELISA (*n* = 3).

### 4.7. Cell Culture 

The THP-1 cell line was kindly provided by Dr. Nuno Santos (CBME, University of Algarve, Faro) and cells were cultured according to ATCC instructions in the RPMI Growth Medium (RPMI 1640 with L-Glutamine (Lonza, Basel, Switzerland), 10% heat-inactivated fetal bovine serum (FBS, Invitrogen, Waltham, MA, USA) and 1% Pen-Strep (P/S, Gibco, Waltham, MA, USA). Differentiation into macrophagic THP-1 (THP-1 MoM) was achieved by culturing cells in 25 ng/mL PMA (Sigma Aldrich, Burlington, MA, USA) in complete RPMI for 48 h. 

Human aortic VSMCs (VSMC) were derived from tissue explants as described previously [45], and used between passages 4 and 12. VSMCs were maintained in M199 medium (Life Technologies, Carlsbad, CA, USA) supplemented with 10% fetal bovine serum (FBS) and 1% (*v*/*v*) of P/S.

The primary human articular chondrocytes were commercially acquired (Lonza, Visp, Switzerland), and cultured in advanced Dulbecco’s modified eagle’s medium (Adv DMEM) (Invitrogen, Carlsbad, CA, USA) supplemented with 10% (*v*/*v*) of heat-inactivated FBS, 1 mM of L-Glutamine (L-Gln, Invitrogen, Waltham, MA, USA) and 1% (*v*/*v*) of P/S. 

All cell cultures were maintained at 37 °C in a humidified atmosphere containing 5% CO_2_, and experiments were performed on confluent VSMC and chondrocyte cells, using an average of 1 × 10^6^ cells/mL of THP-1 MoM.

### 4.8. Cellular Proliferation Measurement

Cells were seeded in 96-well plates at 1 × 10^5^ cells/well and cultured in 200 μL of the corresponding cell culture media, supplemented with (11.7 ± 4.5) × 10^9^ particles/mL of each FCNP and FCNG, as quantified by NTA, for 48 h. Cell viability was assessed using the CellTiter 96 cell proliferation assay (Promega, Madison, WI, USA), following manufacturer’s instructions, in triplicate experiments for each cell type (*n* = 3).

### 4.9. Flow Cytometry Analysis

FCNP and FCNG, (11.7 ± 4.5) × 10^9^ particles/mL of each formulation, were incubated with 1 × 10^6^ THP-1 MoM cells in 500 μL media for 2 h. The flow cytometry protocol from ORIGENE was used for cell staining. Briefly, after dethatching, centrifuged cell pellets were blocked with 0.5% BSA for 30 min, incubated with PE anti-mouse/human CD11b antibody (1 µg/mL, Biolegend, San Diego, CA, USA), and fixed in 0.2% PFA in PBS overnight at 4 °C. A permeabilization step with 0.1% Triton in PBS preceded the incubation with the purified polyclonal CTerm-GRP antibody (5 μg/mL (GenoGla Diagnostics, Faro, Portugal)). At the end, the pellets were treated with fluorochrome (ALEXA Fluor^®^ 647 Donkey anti-rabbit IgG Antibody, Biolegend, 1 µg/mL), and resuspended in PBS for analysis on a FACSCalibur Flow Cytometer (BD Biosciences), using Cell Quest Pro 6.0 software. Compensation was performed using single-stain controls and all gates were set based on Fluorescence Minus One (FMO) controls. For each sample 10,000 events were recorded. Flow cytometry data were analyzed using FlowJo software (version 6.7).

### 4.10. Inflammatory Assays

The anti-inflammatory potential of FCNP and FCNG was evaluated in 1 × 10^6^ THP1-MoM cells and confluent primary VSMCs and chondrocytes, plated in 24-well plates with 500 μL media, by pre-treatment of the cells with (11.7 ± 4.5) × 10^9^ particles/mL of each formulation resuspended in the corresponding cell culture media for determined durations, followed by inflammation stimulation with LPS (100 ng/mL), TNFα (20 ng/mL), and IL-1β (10 ng/mL), respectively, without media exchange for an additional 24 h. In some experiments, cells were also pre-treated with dexamethasone (DXM, 2 µM) as the positive anti-inflammatory control. Cell culture media were collected for the quantification of pro-inflammatory cytokines by ELISA, and cells were harvested for total RNA and protein extraction.

### 4.11. Total Protein Extraction

Total protein extracts from THP1-MoM and VSMCs were obtained with RIPA buffer (50 mM Tris HCl pH 8, 150 mM NaCl, 1% NP-40, 0.5% sodium deoxycholate, 0.1% SDS) for 4 h at 4 °C, with constant shaking. RIPA extracts were centrifuged at 16,000× *g* for 30 min at 4 °C. Protein quantification was performed with the MicroBCA protein assay kit (Pierce).

### 4.12. ELISA Assays

The collected cell culture media were centrifuged at 16,000× *g* for 20 min at 4 °C to remove cellular debris, and used for the quantification of TNFα and IL-6 by ELISA (Peprotech, East Windsor, NJ, USA), following the manufacture’s protocols. Cell culture media and total protein extracts were used for GRP quantification by ELISA, as described [11]. 

### 4.13. Electrophoresis and Western Blot

Aliquots of 20 μg of total protein were size-separated on 4–12% (*w*/*v*) gradient polyacrylamide precast gels containing 0.1% (*w*/*v*) SDS (NuPage, Invitrogen, Waltham, MA, USA) and transferred onto a nitrocellulose membrane (Bio-Rad, Hercules, CA, USA) as previously described [5,10,11]. The detection of NFkB and GAPDH was performed by overnight incubation with anti-NFkB p65 (1 μg/mL, Invitrogen) and anti-GAPDH (1:500, Santa Cruz Biotechnology, Dallas, TX, USA). Immunodetection was achieved using species-specific secondary horseradish peroxidase-conjugated antibodies and Western Lightning Plus-ECL (PerkinElmer Inc., Waltham, MA, USA). Image acquisition was performed using an IQ LAS 4000 mini biomolecular imager.

### 4.14. RNA Extraction, cDNA Amplification and Quantitative Real-Time PCR (qPCR)

Total RNA was isolated from cell cultures using the Direct-zol RNA Miniprep kit (Zymo Research, Irvine, CA, USA), according to the manufacturer’s instructions. The RNA concentration was determined by spectrophotometric analysis at 260 nm using a Nanodrop spectrophometer (Thermo Scientific, Waltham, MA, USA). Five hundred nanograms of total RNA were treated with RQ1 RNase-free DNase (Promega, Madison, WI, USA) and reverse-transcribed using Moloney–murine leukemia virus reverse transcriptase (MMLV-RT, Invitrogen), RNase Out (Invitrogen), and an oligo(dT) adapter (ACGCGTCGACCTCGAGATCGATG(T)13), according to the manufacturer’s recommendations. Quantitative PCR was performed with a CFX connect, Real Time System (Bio-Rad, Richmond, CA, USA), SoFast Eva Green Supermix (Bio-Rad, Richmond, CA, USA) and the specific human primer sets GAPDH_1F (5′-AAGGTGAAGGTCGGAGTCAACGGA-3′) and GAPDH_1R (5′-TCGCTCCTGGAAGATGGTGATGGG-3′) to amplify GAPDH; 18S_1F (5′-GGAGTATGGTTGCAAAGCTGA-3′) and 18S_1R (5′-ATCTGTCAATCCTGTCCGTGT-3′) to amplify 18S; GRP_1F (5′-GTCCCCCAAGTCCCGAGATGAGG-3′) and GRP_1R (5′-CCTCCACGAAGTTCTCAAATTCATTCC-3′) to amplify GRP; IL-1β_1F (5′-TGGACAAGCTGAGGAAGATGCTGGT-3′) and IL-1β_1R (5′-CCCTGGAGGTGGAGAGCTTTCAGTT-3′) to amplify IL-1β; IL-6_1F (5′-AAGCAGCAAAGAGGCACTGGCAGAA-3′) and IL-6_1R (5′-CTGCACAGCTCTGGCTTGTTCCTCAC-3′) to amplify IL-6; NFkB_1F (5′-GCAATCATCCACCTTCATTCTCAACTT-3′) and NFkB_1R (5′-CCTCCACCACATCTTCCTGCTTAG-3′) to amplify NFkB; IL-8_1F (5′-CTGCAGCTCTGTGTGAAGGTGCAGT-3′) and IL-8_1R (5′-GCACCCAGTTTTCCTTGGGGTCCAG-3′) to amplify IL-8. Fluorescence was measured at the end of each extension cycle in the FAM-490 channel, and melting profiles of each reaction were constructed to check for unspecific product amplification. Levels of gene expression were calculated using the comparative method (ddCt) and normalized using gene expression levels of 18S and GAPDH housekeeping gene, for THP1-MoM and VSMCs, respectively, with the iQ5 software (Bio-Rad); qPCR was performed via three independent experiments, each in triplicate (*n* = 3), and a normalized SD was calculated.

### 4.15. Statistical Analysis

Statistical analysis was performed using PRISM software (GraphPad Prism, GraphPad Software, La Jolla, CA, USA). Data are presented as mean ± standard deviation (SD). Student’s *t*-test was used for comparison between two groups. For more than two groups, significance was determined using ordinary one-way ANOVA with comparison between groups by Dunnett test. Statistical significance was defined as *p* ≤ 0.05 (*), *p* ≤ 0.01 (**), *p* ≤ 0.001 (***) and *p* ≤ 0.0001 (****).

## 5. Patents

The tools and methods described in this manuscript are included in a PCT patent application, PCT/PT2009000046.

## Figures and Tables

**Table 1:** Physicochemical properties of fluorescein-labeled quitosan–tripolyphosphate nanoparticles (FCNP) and fluorescein-labeled quitosan–ucGRP–tripolyphosphate nanoparticles (FCNG) assessed by DLS (*n* = 3).

	Size (nm)	PDI	Zeta Potential (mV)	AE (%)
FCNP	130 ± 45	0.33 ± 0.07	+37 ± 2	
FCNG	156 ± 38	0.39 ± 0.06	+28 ± 7	99.8 ± 0.1

DLS, dynamic light scattering; PDI, polydispersion index; AE, association efficiency.

## Data Availability

Not applicable.
